# Carotenoids and Cognitive Outcomes: A Meta-Analysis of Randomized Intervention Trials

**DOI:** 10.3390/antiox10020223

**Published:** 2021-02-02

**Authors:** Sergio Davinelli, Sawan Ali, Vincenzo Solfrizzi, Giovanni Scapagnini, Graziamaria Corbi

**Affiliations:** 1 Department of Medicine and Health Sciences “V. Tiberio”, University of Molise, 86100 Campobass, Italy; s.ali@studenti.unimol.it (S.A.); giovanni.scapagnini@unimol.it (G.S.); graziamaria.corbi@unimol.it (G.C.); 2 Department of Interdisciplinary Medicine, Clinica Medica “Frugoni” and Geriatric Medicine-Memory Unit, University of Bari Aldo Moro, 70124 Bari, Italy; vincenzo.solfrizzi@uniba.it

**Keywords:** carotenoids, cognition, diet, antioxidants, neuroprotection, prevention

## Abstract

Recent evidence suggests that diet can modify the risk of future cognitive impairment and dementia. A biologically plausible rationale and initial clinical data indicate that the antioxidant activities of dietary carotenoids may assist the preservation of cognitive function. A meta-analysis of randomized controlled trials was conducted to examine the relationship between carotenoid supplementation and cognitive performance. A literature search was conducted in the MEDLINE (via PubMed), Scopus, Web of Science, and Cochrane databases from their inception to July 2020. A total of 435 studies were retrieved. Abstract screening using predefined inclusion and exclusion criteria was followed by full-text screening and data extraction of study characteristics and measured outcomes. A meta-analysis of eligible trials was performed using a random-effects model to estimate pooled effect size. We identified 9 studies with a total of 4402 nondemented subjects, whose age ranged from 45 to 78 years. Results of the pooled meta-analysis found a significant effect of carotenoid intervention on cognitive outcomes (Hedge’s g = 0.14; 95% confidence interval: 0.08, 0.20, *p* < 0.0001). There was no evidence of heterogeneity among the studies (τ2 = 0.00, I^2^ = 0.00%, H^2^ = 1.00) or publication bias. Although further studies are needed, our results suggest that carotenoid interventions are associated with better cognitive performance. Thus, these dietary compounds may help to reduce the risk of cognitive impairment and dementia.

## 1. Introduction

Cognitive function encompasses a wide array of mental abilities, including learning, thinking, reasoning, remembering, problem solving, decision making, and attention. With aging, several cognitive domains associated with processing speed, working memory, and executive processes gradually decline, becoming slower and inefficient [1,2]. The brain, with its high oxygen consumption and lipid-rich content, is highly susceptible to oxidative stress. Therefore, long-term oxidative damage has a strong potential to negatively impact cognitive abilities [1]. In addition to oxidative injury, cognitive deterioration is the result of multiple and overlapping mechanisms, including neuroinflammation, neural cell loss, and hypofunction of the monoaminergic and cholinergic pathways [2,3,4]. Accumulating evidence indicates that many of the underlying neuropathological mechanisms associated with dementia begin a decade or more before the onset of symptoms [5,6]. Therefore, the identification of specific interventions that may prevent the decline in cognitive function during this preclinical phase is crucial in terms of public health policy.

Non-pharmacological approaches focused on lifestyle factors represent a cost-effective and practical strategy to reducing or slowing age-related cognitive decline [7]. Nutrition is a modifiable lifestyle factors that has been consistently associated with cognition at various levels, including greater adherence to healthy dietary patterns, the intake of specific nutrients, or the consumption of specific foods [8,9,10]. As summarized in recent reviews, results from both observational and intervention studies show that a wide variety of dietary antioxidants could affect the development of cognitive impairment [11,12,13].

Carotenoids belong to a large family of fat-soluble plant pigments that are widely present in yellow-orange vegetables and fruits, such as carrots, apricots, cantaloupe, and tomatoes [14]. This class of phytochemicals is classified into two groups, namely carotenes and xanthophylls. As carotenoids cannot be synthesized de novo by humans, we obtain them exclusively from the diet or via supplementation. Of more than 700 carotenoids, 6 are commonly found in the human diet and serum: α-carotene, β-carotene, lutein, zeaxanthin, lycopene, and β-cryptoxanthin [15]. Recently, there has also been a growing interest in the clinical application of astaxanthin, a xanthophyll carotenoid synthesized by a number of bacteria, microalgae, and yeasts [16]. Overall, the consumption of these dietary carotenoids has been associated with various health benefits, including a reduced risk of age-related macular degeneration (AMD), some cancers, and coronary heart disease (CHD) [17].

Some carotenoids, such as lutein and zeaxanthin, can cross the blood–brain barrier, reach the brain and accumulate in the macular pigment of the retina [18,19]. Lutein, in particular, is known to accumulate across all cortices and brain membranes. In contrast, β-carotene, β-cryptoxanthin, and α-carotene are generally not found in the retina, but the serum concentrations of these carotenoids can be used as a biomarker to predict brain carotenoid concentrations [18,19,20,21]. There is mounting evidence that carotenoids may positively affect cognitive function through their antioxidant and anti-inflammatory effects [21,22,23]. However, these compounds may act more specifically in the neural circuits by increasing neural efficiency or stabilizing lipid-protein structures in neuronal membranes. Other neuroprotective mechanisms by which certain carotenoids may influence interneuronal function include the enhancement of the gap junctional communication and the modulation of the functional properties of synaptic membranes [24,25,26].

To date, findings regarding the possibility that supplementation with carotenoids may delay the onset of cognitive decline or even ameliorate cognitive performance remain inconsistent across reviews. There is currently no published meta-analysis to our knowledge that provides a quantitative measure of the association between carotenoids and cognitive function. Therefore, a systematic review and meta-analysis was conducted to evaluate the potential clinical effects of carotenoids on cognition in human adults.

## 2. Methods

This systematic review with meta-analysis was conducted in accordance with established guidelines from Preferred Reporting Items for Systematic Reviews and Meta-Analysis (PRISMA) [27].

### 2.1. Search Strategy

We searched the PubMed, Scopus, Cochrane Library, and Web of Science databases to identify randomized clinical trials (RCTs) published in peer-reviewed journals. In order to conduct a comprehensive systematic literature search, we used both controlled vocabulary and free text terms. The search was conducted using Boolean operators “AND” and “OR” to combine the following terms: “α-carotene” OR “alpha-carotene” OR “β-carotene” OR “beta-carotene” OR “β-cryptoxanthin” OR “beta-cryptoxanthin” OR “lutein” OR “zeaxanthin” OR “lycopene” OR “astaxanthin” AND “cognitive function” OR “dementia” OR “Alzheimer’s disease” OR “vascular dementia” OR “dementia with Lewy body” OR “Lewy body dementia” OR “frontotemporal dementia” OR “cognitive decline” OR “cognitive impairment” OR “cognitive loss” OR “cognition” OR “memory” OR “mild cognitive impairment” OR “attention” OR “reaction time” OR “speed of processing” OR “crystallized ability” OR “crystallized intelligence” OR “fluid ability” OR “fluid intelligence” OR “general mental ability” OR intelligence OR “executive function” OR “neuropsychological test” OR “neuropsychological assessment” OR “mini mental state examination” AND “clinical trial” OR “intervention study”. At the same time, similar queries were respectively used for controlled vocabulary search: “carotenoids” [Mesh] AND “cognition” [Mesh], INDEXTERMS “cognition” AND INDEXTERMS “cognition”.

### 2.2. Eligibility Criteria

Clinical studies, which clearly assessed the effect of carotenoids on the cognitive function, published up to 31 July 2020, were included in the review. Clinical trials that fulfilled the following criteria were eligible: the design was a randomized clinical trial; studies that reported the carotenoid content of foods or the dose of the carotenoid-containing supplements; studies that were conducted in adult participants (≥18 years old) with or without mental disorders; studies that provided sufficient information about cognitive outcomes before and after the intervention in the active and control group. Articles were excluded from the review for the following reasons: studies not published in English; articles that used secondary data such as reviews, meta-analysis, conference papers and book chapters; studies on animal models or in vitro experiments; studies that did not allow the extraction of cognitive scores.

### 2.3. Selection Process and Data Extraction

Two authors (SD and SA) independently screened the titles and abstracts of all the articles retrieved to identify those that met the eligibility criteria. The full texts of the remaining studies were then retrieved and independently assessed by the same authors. In the case of disagreement about the eligibility of a study, a third author (GC) decided which articles were included. Data from all included articles were extracted by one author (SA) and checked by two authors (SD and GC). The following information was recorded: author’s name, publication year, study country, study design, participant characteristics (sample size, gender, and age), health status, procedures used to measure cognitive outcomes, intervention (type of compounds and dose), and results.

### 2.4. Quality Assessment

Two authors (SA and SD) independently assessed the study quality and risk of bias of each included study, using the Cochrane risk of bias tool for RCTs [28]. The risk of bias was assessed as being ‘low’, ‘high’ or ‘unclear’ across the following domains: (1) sequence generation, (2) allocation sequence concealment, (3) blinding of participants and personnel, (4) blinding of outcome assessment, (5) incomplete outcome data, and (6) selective outcome reporting. Discrepancies in the risk of bias assessment were resolved by discussion among review authors.

### 2.5. Statistical Analysis

The data from the included studies were expressed as mean difference and SD with a 95% confidence interval. Participants who consumed the carotenoid intervention were recorded as the treated group, while those who consumed the placebo intervention were recorded as the control group. The summary statistics required for each outcome were the number of participants in the active and control groups at post-test, and the mean and SD of the cognitive outcome after the intervention. Random-effects models were prespecified a priori, given the heterogeneity across interventions, participants, and assessment of outcomes [29]. Heterogeneity was evaluated using Chi-squared test, and any *p* value ≤ 0.05 was considered statistically significant. Inconsistency was examined using I^2^ and the following grades were applied: <25% (very low), 25 to <50% (low), 50 to <75% (moderate), and ≥75% (large) [30,31]. If the heterogeneity was small or absent, a fixed effects model was applied for the meta-analysis. In sensitivity analyses, we excluded studies whose trial sample size affected the overall estimate or heterogeneity. The visual inspection of funnel plots and Egger’s test were used to assess the publication bias [32,33]. The meta-analysis was carried out using Stata 16 software package (STATA Corp., College Station, TX, USA). Two-sided *p* value ≤ 0.05 was considered statistically significant.

## 3. Results

### 3.1. Search Results

**Figure 1:**
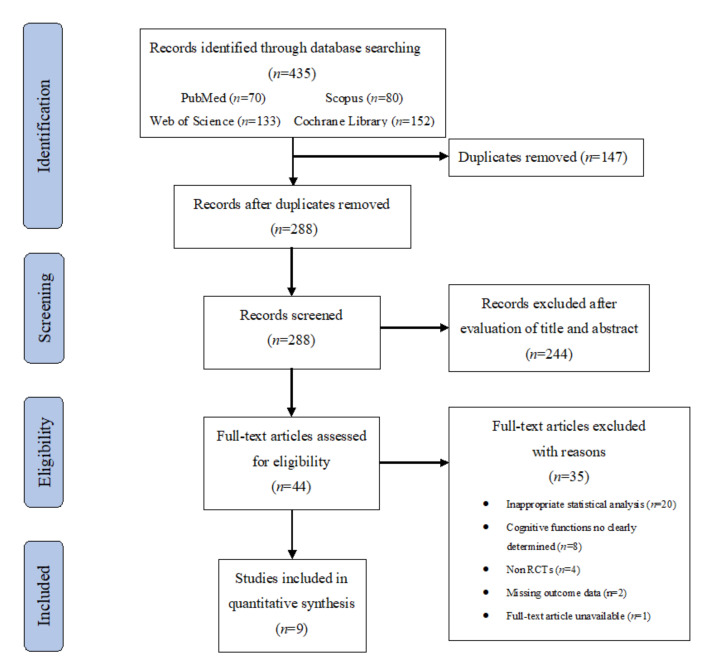
Flowchart of search results and study retrieval.

The flow of records through the review is summarized in Figure 1. The combined search from four databases resulted in 435 potentially appropriate publications. After removing duplicates, 288 records were screened by title and abstract, which resulted in the exclusion of 244 records. The full texts of the remaining 44 articles were examined for eligibility assessment, after which 35 studies were excluded for the following reasons: inappropriate statistical analysis (*n* = 20); cognitive functions not clearly determined (*n* = 8); non RCTs (*n* = 4); missing outcome data (*n* = 2); full-text article unavailable (*n* = 1). Thus, a total of 9 studies [34,35,36,37,38,39,40,41,42] were included in the final analyses.

### 3.2. Study Characteristics

Table 1 summarizes the characteristics of the included studies that addressed the effect of carotenoid intervention on cognitive function. The studies were published from 2007 to 2018 and were conducted in 3 different countries: United States, Japan, and Ireland. A total of 4402 individuals were randomly assigned to receive carotenoid intervention or placebo. Grodstein et al. enrolled the highest number of participants (*n* = 4052) [34]. Seven trials were conducted in both sexes [35,36,37,39,40,41,42], whereas 2 studies were exclusively conducted in males or females [34,38]. The age of all participants varied from 45 to 78 years. All the trials measured cognitive function in healthy subjects, except for one study that focused on participants affected by mild cognitive impairment (MCI) [37]. A wide variety of neurocognitive test batteries were used in the included studies, such as CNS Vital Signs [35], Mini-Mental State Examination (MMSE) [34], CogHealth, Groton Maze Learning Test (GMLT) [39], and Cambridge Neuropsychological Test Automated Battery (CANTAB) [42]. The dosage of carotenoids administered in the studies ranged from 0.5 mg/d to 50 mg/d. The majority of clinical trials assessed the effect of xanthophylls such as lutein, zeaxanthin, and astaxanthin [35,36,37,38,39,40,41,42], whereas only one study determined the effects of β-carotene [34]. The duration of carotenoid supplementation ranged from 2 weeks to 12 months. However, Grodstein et al. conducted a long-term β-carotene intervention with a mean treatment duration of 18 years [34]. All studies were randomized, double-blinded, placebo-controlled trials, except the study by Scott et al. which was not a double-blinded trial [42]. 

### 3.3. Quality Assessment

**Figure 2:**
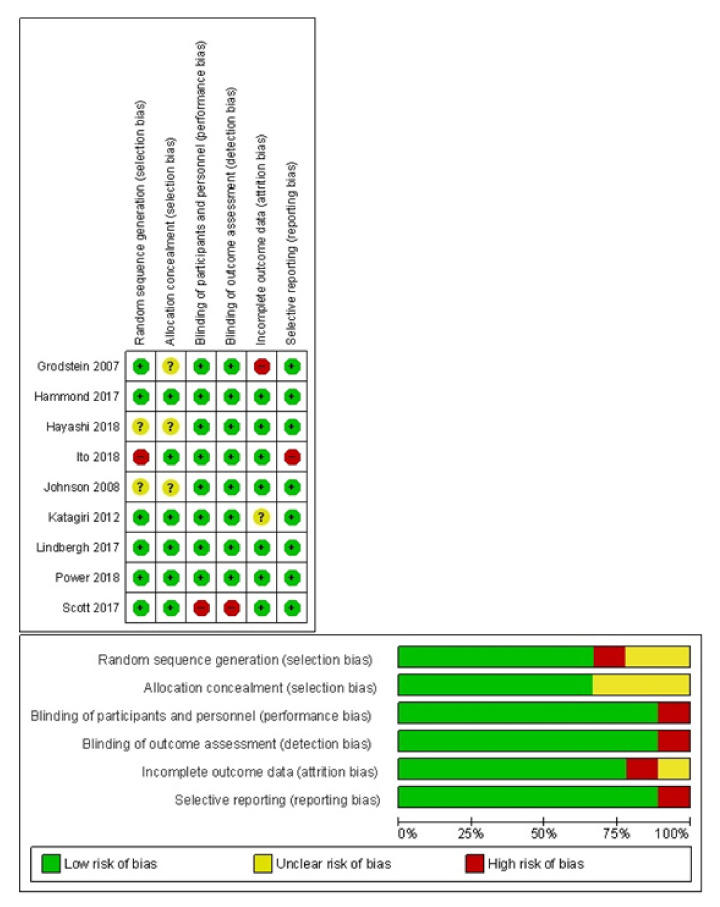
Risk of bias assessment for the included randomized controlled trials. Review of authors’ judgement about each risk of bias item presented as percentages across all included studies.

The risk of bias assessment is summarized in Figure 2. The majority of studies (66%) described the process of sequence generation or allocation concealment in sufficient detail and were judged as having a ‘low’ risk of bias for these domains. Eight out of the 9 clinical trials reported blinding of participants, personnel, and outcome assessment. If studies did not employ intention-to treat principles in the data analysis or did not give the number of dropouts, they were regarded as having a high risk of bias for incomplete outcome data. All other domains were evaluated to have a low to unclear risk of bias.

### 3.4. Meta-Analysis of the Effect of Carotenoids on Cognitive Outcomes

**Figure 3:**
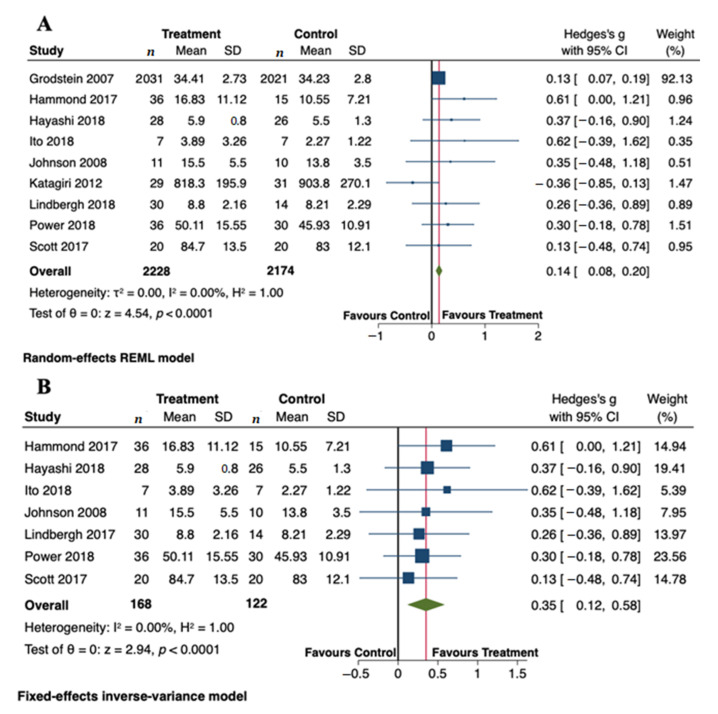
Forest plots summarizing the association between carotenoid intervention and cognitive outcomes. (**A**) Primary analysis using a random-effects model (**B**) Sensitivity analysis using a fixed-effects model. Pooled summary data are presented as mean difference compared to control. The size of the data markers indicates the weight assigned to each study in the meta-analysis. Squares represent the standard mean differences, bars represent the 95% CI, and diamonds represent the pooled analysis.

**Figure 4:**
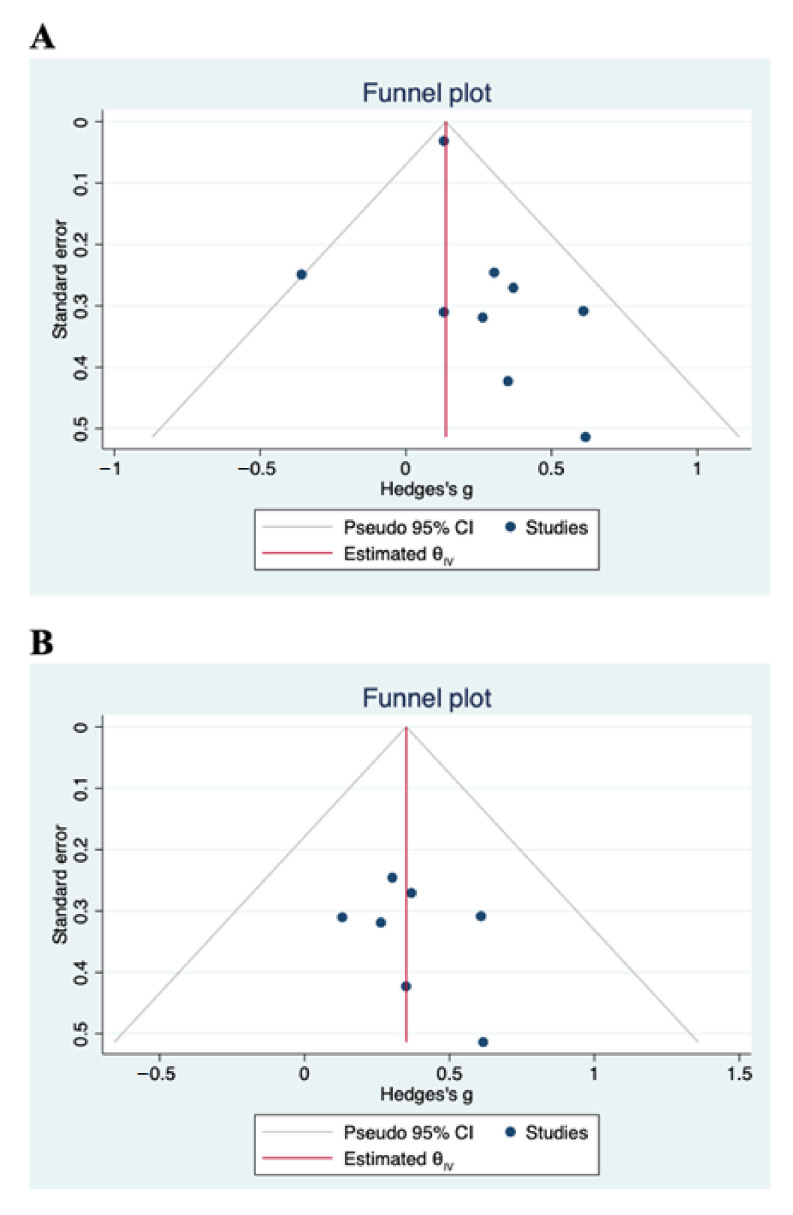
Funnel plots with pseudo-95% CIs for publication bias of (**A**) primary analysis and (**B**) sensitivity analysis. Visual inspection indicates no evidence of publication bias.

Figure 3A displays a forest plot with the summary effect of carotenoid intervention on cognitive functions. Overall, we meta-analyzed 9 RCTs involving 2228 and 2174 subjects in the treated and control groups, respectively. Meta-analysis showed a statistically significant improvement of cognitive functions after administration of carotenoids (Hedge’s g = 0.14; 95% CI: 0.08, 0.20, *p* < 0.0001). Although there was no evidence of significant heterogeneity among studies (τ2 = 0.00, I^2^ = 0.00%, H^2^ = 1.00), we carried out a sensitivity analysis by omitting two studies from the pooled analysis. We removed the study by Grodstein et al. [34] because its relative contribution to the pooled meta-analysis result (weight = 92.13%) obscured the contribution of each study to the overall result. We also excluded the study by Katagiri et al. [39], mainly because of a higher degree of heterogeneity among studies. Using a fixed-effects model, the sensitivity analysis did not change the overall findings obtained from the primary analysis. The positive effects of carotenoids on cognitive functions remained significant (Hedge’s g = 0.35; 95% CI: 0.12, 0.58, *p* < 0.0001) (Figure 3B). The total number of subjects in the sensitivity analysis was 290. The absence of statistical heterogeneity has also been confirmed (τ2 = 0.00, I^2^ = 0.00%, H^2^ = 1.00). We present the funnel plots for the primary and sensitivity analyses in Figure 4. Visual inspection of the funnel plots and Egger’s test (*p* = 0.789) indicate no evidence of asymmetry, and, therefore, no strong evidence of publication bias.

## 4. Discussion

Along with exercise and social activities, nutrition has important roles in preserving cognition and reducing the risk of dementia. Age-related cognitive decline can be due to failure of protective mechanisms caused by dietary deficiencies, for instance in antioxidants such as carotenoids. Epidemiological studies evaluating the relationship between dietary carotenoids and maintenance of cognition reported that low carotenoids levels could play a role in cognitive impairment [43,44]. To date, few clinical trials have investigated the relationship between dietary carotenoids and cognitive performance. However, the purpose of the present review was to summarize the available clinical evidence and determine whether carotenoid supplementation has the potential to be used as a nutritional strategy for cognitive maintenance.

Our meta-analysis pooled results from 9 clinical trials to estimate the effects of the administration of carotenoids on outcomes associated with cognitive functions. In the overall analysis, we found that carotenoid supplementation may help to improve cognitive performance in relatively healthy participants aged 45–78 years. Our findings are partially in agreement with previous narrative reviews indicating that higher dietary intake and blood concentrations of carotenoids are associated with lower risk of age-related cognitive dysfunction [45,46,47]. It is indeed known that low concentrations of dietary carotenoids may precede or be a consequence of cognitive impairment [48,49].

Although the underlying neuroprotective mechanism of carotenoids is not clear, this may be a result of their antioxidant, anti-inflammatory, and anti-apoptotic effects, as well as the potential to promote or maintain neural plasticity [50,51]. Despite the small number of publications included in this meta-analysis, according to our findings, it is reasonable to design more large-scale randomized trials with long-term treatment to determine whether and to what extent dietary carotenoids may be used as neuroprotective agents to preserve neural resources throughout the lifespan. Most of the trials examined here used different treatment durations and involved a large variety of dosages and sources of carotenoids. Therefore, the results from these clinical studies do not provide a clear picture of the optimal dosage and treatment duration. Lutein and zeaxanthin were the most frequently tested carotenoids in the included clinical trials. These studies indicate that the administration of lutein or lutein plus zeaxanthin may improve cognitive effects in older men and women. Women who received lutein 12 mg/d for 4 months improved verbal fluency score [38]. Men and women who received lutein 12 mg plus zeaxanthin 2 mg for 12 months improved domains of the CNS Vital Signs test battery, such as complex attention, cognitive flexibility, and composite memory [40]. In another study not included here, Walk et al. showed that a higher macular pigment optical density (MPOD), a surrogate measure of lutein and zeaxanthin concentration in the brain, is correlated with a better cognitive performance and better neural efficiency in preadolescent children [52]. These findings suggest that lutein and zeaxanthin may be also effective in young subjects. Three of the 9 articles identified for inclusion in the present review reported significant results of astaxanthin on cognitive functions. One trial used low and high doses (6 and 12 mg/d) of astaxanthin for 12 weeks in middle-aged and elderly subjects who complained age-related forgetfulness. Cognitive performance improved in both low and high dose groups [39]. Similar results were also observed in subjects treated with astaxanthin doses of 8 and 6 mg/d for 2 and 3 months, respectively [36,37]. Besides the well-known antioxidant effect, astaxanthin may offer neuroprotection and improve cognitive functions by promoting neurogenesis through modulating microglial activity and important signaling molecules such as extracellular signal-regulated kinase (ERK) and brain-derived neurotrophic factor (BDNF) [53,54].

Although we found a significant improvement in cognitive performance associated with carotenoid administration, it is important to point out that our meta-analysis was heavily influenced by the large number of participants involved in the study of Grodstein et al. [34]. This randomized trial represents a landmark study on the topic, and its inclusion was warranted. However, the sensitivity analysis restricted to 7 studies revealed that the pooled result was robust. Some methodological considerations should also be highlighted. First, many different types of cognitive batteries and tests were used. Despite this, a visual inspection of funnel plot indicates that there is no heterogeneity among the studies. Second, the beneficial effects of carotenoids may depend on their bioavailability and this is influenced by absorption, metabolism, and disposition in brain tissue. The rate of absorption was not measured in most included studies and this aspect should be considered when interpreting the results.

To our knowledge, this is the first systematic review and meta-analysis to specifically investigate the effects of carotenoids on outcomes associated with cognitive performance. We included only randomized controlled trials which are considered the ‘gold standard’ of clinical studies. A quality assessment of clinical trials was performed, and most of the included studies had a relatively good quality. Furthermore, no evidence of publication bias was seen. The literature search was conducted applying specific eligibility criteria, using controlled vocabulary queries, and, therefore, the authors are confident that the search strategy retrieved all relevant studies.

Although the results of this meta-analysis are promising for conducting future clinical trials in this research area, there are several limitations that need to be acknowledged. The main limitation is the small number of included studies. Except for one trial, the number of subjects in each study was small, and therefore our findings must only be viewed as preliminary. The small number of included studies also prevented subgroup analysis and meta-regression. Moreover, no clinical study investigated the effects α-carotene, β-cryptoxanthin, and lycopene on cognitive function.

## 5. Conclusions

In conclusion, these results highlight the potential role of carotenoids in the protection of mental functions even in subjects without cognitive impairment. This is particularly important because the population is aging, and preservation of cognitive function is crucial for individual autonomy and quality of life, even in non-demented subjects. However, our findings should be interpreted cautiously, and further well-powered and long-term trials are required to determine treatment duration, type of carotenoid, and optimal dosage.

## Figures and Tables

**Table 1:** Overview of studies investigating the relationship between carotenoids and cognitive outcomes.

Reference	Location	Population Characteristics and Study Design	Intervention	Outcome of Interest	Cognitive Test Used	Results
Grodstein et al. (2007) [34]	USA	*n* = 4052 M (mean age 55.9 years) Condition: Healthy subjects Design: Randomized, double-blind, placebo-controlled trial Duration: 18 years	β-carotene (50 mg on alternate days)	Global cognition Verbal memory Category fluency	TICS (MMSE) EBMT CFT	Improvement in global cognitive score (*p* = 0.03), verbal memory (*p* = 0.007), and TICS score (*p* = 0.04)
Hammond et al. (2017) [35]	USA	*n* = 51 (21 M and 30 F; mean age 73.7 years) Condition: Healthy subjects Design: Randomized, double-blind, placebo-controlled trial Duration: 12 months	Lutein + zeaxanthin (12 mg/d; 10 mg of lutein and 2 mg of zeaxanthin)	Verbal memory Visual memory Reasoning ability Executive function Psychomotor speed Complex attention Cognitive flexibility Global cognition	CNS Vital Signs	Improvement in complex attention (*p* < 0.02), cognitive flexibility (*p* < 0.04) ,and composite memory in male (*p* = 0.04)
Hayashi et al. (2018) [36]	Japan	*n* = 54 (25 M and 29 F, age range 45–64 years) Condition: Healthy subjects Design: Randomized, double-blind, placebo-controlled trial Duration: 2 months	Astaxanthin (8 mg/d)	Immediate and short-term memory Memory-recall ability Cognitive interference inhibition	Word memory test Verbal fluency test Stroop test	Improvement in immediate recall (*p* < 0.05), recall after 5 min + cued recall (*p* < 0.05), verbal fluency (*p* < 0.01), and Stroop test score (*p* < 0.01)
Ito et al. (2018) [37]	Japan	*n* = 14 (9 M and 5 F; age range 57–78 years) Condition: MCI Design: Randomized, double-blind, placebo-controlled trial Duration: 3 months	Astaxanthin + sesamin (16 mg/d; 6 mg of astaxanthin)	Composite memory Verbal memory Visual memory Processing speed Psychomotor speed Executive function Reaction attention Complex attention Simple attention Cognitive flexibility Motor speed	CNS Vital Signs ADAS-Cog	Improvement in psychomotor speed (*p* < 0.05) and processing speed (*p* < 0.05)
Johnson et al. (2008) [38]	USA	*n* = 21 F (mean age 67.3 years) Condition: Healthy subjects Design: Randomized, double-blind, placebo-controlled trial Duration: 4 months	Lutein (12 mg/d)	Memory Processing speed Attention	Verbal fluency test Digit span forward Digit span backward MIR apartment test Shopping list test Pattern recognition test	Improvement in verbal fluency scores (*p* < 0.03)
Katagiri et al. (2012) [39]	Japan	*n* = 60 (M & F; mean age 51.5 years) Condition: Healthy subjects with age-related forgetfulness Design: Randomized, double-blind, placebo-controlled trial Duration: 3 months	Astaxanthin (high dosage group 12 mg/d)	Response time Accuracy Spatial working memory	CogHealth battery GMLT	Improvement in working memory (*p* = 0.044), delayed recall score (*p* = 0.028), and improvement of GMLT total errors (*p* < 0.01)
Lindbergh et al. (2018) [40]	USA	*n* = 44 (M 18 and F 26; mean age 72 years) Condition: Healthy subjects Design: Randomized, double-blind, placebo-controlled trial Duration: 12 months	Lutein + zeaxanthin (12 mg/d; 10 mg of lutein and 2 mg of zeaxanthin)	Verbal learning	fMRI-adapted learning Word recall tests	No effect in word recall score (*p* > 0.05). Improvement in verbal learning (*p* < 0.05)
Power et al. (2018) [41]	Ireland	*n* = 66 (M & F; mean age 46.4 years) Condition: Healthy subjects Design: Randomized, double-blind, placebo-controlled trial Duration: 12 months	Carotenoids (22 mg/d; 10 mg of lutein, 10 mg of meso-zeaxanthin, and 2 mg of zeaxanthin)	Phonemic fluency	FAS test	Improvement in phonemic fluency (*p* < 0.001)
Scott et al. (2017) [42]	USA	*n* = 40 (M 15 and F 25, mean age 63.3 years) Condition: Healthy subjects Design: Randomized, placebo-controlled trial Duration: 6 months	Lutein (0.5 mg/d)	Attention Visual memory Executive function Working memory Planning	CANTAB	Improvement in sustained attention (*p* = 0.033), memory (*p* = 0.001), and spatial working memory (*p* = 0.032)

Abbreviations: TICS, Telephone Interview for Cognitive Status; MMSE, Mini-Mental State Examination; EBMT, The East Boston Memory Test; CFT, Category Fluency Test; MCI, Mild Cognitive Impairment; ADAS-Cog, Alzheimer’s Disease Assessment Scale-Cog; GMLT, the Groton Maze Learning Test; fMRI, Functional Magnetic Resonance Imaging; FAS, Phonemic verbal fluency; CANTAB, Cambridge Neuropsychological Test Automated Battery.
